# Objective Analysis of Movement Behaviours and Cardiometabolic Health in Sub‐Saharan Africans Living in the UK: A Cross Sectional Study

**DOI:** 10.1002/hsr2.72618

**Published:** 2026-06-12

**Authors:** Damilola A. Ibirogba, Luis S. Andalco, Matteo Crotti, Sarah J. Charman, Amy S. Fuller, Titilope Ajepe, Michael Duncan, Alasdair P. Blain, Faatihah Niyi‐Odumosu, Peter W. Mwangi, Federick O. Bukachi, Olufumilola L. Dominic, Otto F. Barak, Djordje G. Jakovljevic, Nduka C. Okwose

**Affiliations:** ^1^ Clinical Sciences and Translational Medicine Research Group, Centre for Discoveries in Life Sciences Coventry University Coventry UK; ^2^ Sigma Statistics Service Coventry University Coventry UK; ^3^ Department of Human and Social Sciences University of Bergamo Bergamo Italy; ^4^ Translational and Clinical Research Institute Newcastle University Newcastle Upon Tyne UK; ^5^ Department of Physiotherapy Coventry University Coventry UK; ^6^ Centre for Healthcare and Communities Transformation Coventry University Coventry UK; ^7^ Centre for Health and Clinical Research University of the West of England Bristol UK; ^8^ Department of Medical Physiology University of Nairobi Nairobi Kenya; ^9^ Department of Human Kinetics Education University of Ilorin Ilorin Nigeria; ^10^ Faculty of Medicine and Faculty of Sports and Physical Education University of Novi Sad Novi Sad Serbia

**Keywords:** compositional analysis, isotemporal substitution, physical activity, sedentary, sleep

## Abstract

**Background and Aims:**

Movement behaviours, including sedentary behaviour (SB), physical activity (PA), and sleep, play a crucial role in cardiometabolic health. This study evaluated their relationships and assessed the effects of reallocating SB to different PA intensities.

**Methods:**

This cross‐sectional study conducted between March 2024 and March 2025, included 75 individuals from Sub‐Saharan Africa (SSA) (51% female; mean age: 40 ± 10 years). Time (minutes/day) spent in the different movement behaviours was determined using wrist‐worn accelerometers (GENEActiv, UK). Cardiometabolic outcomes included body mass index (BMI), waist circumference (WC), HDL cholesterol, total: HDL cholesterol ratio, random glucose, and cardiorespiratory fitness. Pairwise correlations and compositional analyses were performed to investigate associations between movement behaviours and cardiometabolic outcomes.

**Results:**

The median daily movement composition (minutes) was sleep (346), SB (672), LPA (112), and MVPA (96). MVPA showed a moderate inverse relationship with total cholesterol and was the only movement category associated with cardiorespiratory fitness. Sleep duration showed an inverse relationship with BMI and WC. Reallocating 30 min of SB to sleep, LPA, or MVPA did not result in significant changes in cardiometabolic outcomes (*p* > 0.05 for all comparisons).

**Conclusion:**

Moderate‐to‐vigorous physical activity showed the most consistent associations with favourable cardiometabolic markers, suggesting it may play a particularly important role in cardiometabolic health among this population. However, these benefits may be dependent on prolonged exposures. Sleep regulation may also contribute to the management of adiposity.

## 1 Introduction

Global morbidity and mortality arising from type 2 diabetes mellitus, hypertension, dyslipidaemia, obesity, and atherosclerotic cardiovascular disease, a spectrum of diseases collectively called cardiometabolic disease, continues to increase, achieving epidemic status [1]. Individuals of Black heritage, particularly those in high‐income countries present with earlier onset, greater severity, and increased risk of complications and mortality compared to other ethnic groups [2]. These are attributable to several socioeconomic and structural barriers, including physical inactivity, limited access to safe green spaces, and a lack of culturally tailored health promotion strategies, all of which hinder engagement and/or adherence to healthy movement behaviours [3].

Movement behaviours, defined within the 24‐h paradigm as inclusive of sedentary behaviour (SB), physical activity (PA), and sleep, are now recognised as critical, modifiable determinants of cardiometabolic health [4]. Interacting synergistically, these behaviours influence metabolic, autonomic, and cardiovascular functions when assessed objectively [5]. Several studies have highlighted the relative cardiometabolic effects of reallocating time from one behaviour to another [6, 7, 8]. For example, low PA below WHO recommendations, combined with short (< 6 h) or long (> 10 h) sleep, is associated with increased all‐cause mortality risk in the general population [9]. Moderate‐to‐vigorous intensity physical activity (MVPA) has also been reported as the most important contributor to improved cardiorespiratory fitness and reduced cardiometabolic risk [10]. Similarly, there is a reduction in fasting glucose and higher insulin sensitivity when 30 min of time spent awake sitting/lying, standing, or in light intensity physical activity (LPA) were substituted with MVPA [11].

Kufe et al. (2022) [12] conducted the only known study examining the relationship between movement behaviours and markers of type 2 diabetes risk in individuals from sub‐Saharan Africa (SSA). Their findings indicated that higher total movement volume was associated with lower glycaemic levels and improved insulin sensitivity, though these associations lost significance after adjusting for adiposity. Notably, they reported that replacing 30 min of inactivity or light PA with an equivalent duration of MVPA led to improvements in insulin and glycaemic markers related to type 2 diabetes risk. Despite these benefits, evidence on the associations between objectively assessed movement behaviours in relation to a broad spectrum of cardiometabolic markers, including blood glucose, lipids, and cardiorespiratory fitness is warranted in Black individuals from SSA. The aims of the present study were twofold; (i) to evaluate cross‐sectional associations of SB, PA, and sleep with cardio‐metabolic risk biomarkers in Black adults from SSA resident in the UK, and (ii) to evaluate the impact of reallocating time between 24‐h movement behaviours on cardiometabolic health.

## 2 Methods

### 2.1 Study Design, Participants, and Recruitment

This cross‐sectional prospective study recruited 75 Black adults originally from SSA resident in the West Midlands, UK. On confirmation of eligibility (i.e., no history of: ‘chronic respiratory, cardiovascular, or related conditions, i.e., COPD, emphysema, pulmonary hypertension, and coronary artery disease; severe hypertension; kidney disease; cancer; acute or chronic neurological impairment or progressive neurological disease; or body mass index ≥ 40 kg/m^2^’), participants attended the cardiovascular research laboratory at Coventry University for a single visit.

### 2.2 Study Assessments

Participants were instructed to abstain from alcohol and caffeine and to avoid vigorous physical activity for 24 h prior to testing, and to fast overnight. Anthropometrics, medical history and physical examination, including 12‐lead electrocardiogram and blood pressure were performed on day of recruitment.

### 2.3 Ethical Approval

The study protocol and procedures were in accordance with the Declaration of Helsinki and participants provided written informed consent before taking part in the study. Ethical approval was granted by Coventry University Research Ethics Committee (P169958, January 2024).

### 2.4 Movement Behaviours

To measure changes in free living movement behaviours, participants wore a waterproof tri‐axial, raw data accelerometer (GENEActiv, UK) continuously on the non‐dominant wrist for 7 days. Movement behaviours classified were sleep, SB (time spent sitting or lying outside of sleep), LPA (ambulatory but non‐purposeful walking), and MVPA (walking with cadence ≥ 100 steps/min, running, cycling, inclined stepping). Raw accelerometer data were processed using R‐package GGIR (Version 1.5–21) [13, 14]. Acceleration calibration error was corrected as previously described [15]. The first and last hour of the measurement were excluded as they are expected to be influenced by the monitor distribution and collection procedure [16]. The average magnitude of wrist acceleration per 5‐s epoch was calculated with Euclidean norm minus one (ENMO) metric (1 mg = 0.001 × gravitational acceleration) [14]. Time spent in the following acceleration thresholds was calculated: inactivity (< 40 mg cut‐off), LPA (40–100 mg cut‐off), and MVPA (≥ 100 mg cut‐off) [17]. Total activity time within waking hours was recorded as step count. Estimated total sleep duration, wakefulness after sleep onset (WASO), and sleep efficiency were calculated.

### 2.5 Cardiometabolic Outcomes

Two markers of adiposity were assessed by the research team: body mass index (BMI, kg/m^2^) and waist circumference (WC). Cardiometabolic biomarkers included: total cholesterol (TC), high‐density lipoprotein (HDL), non‐HDL, TC: HDL cholesterol ratio, and random blood glucose (CardioCheck Plus, PTS diagnostics USA). Participants also underwent a peak cardiorespiratory fitness test (VO_2_peak) following a ramped exercise protocol on an upright cycle ergometer following previously reported method [18].

### 2.6 Statistical Analyses

#### 2.6.1 Sample Size and Power Considerations

A formal a priori power calculation using standard closed‐form methods was not performed. However, consistent with methodological recommendations [19, 20], an estimation‐focused approach was adopted, and a sample size (*n* = 75) was determined pragmatically based on feasibility and its adequacy to fit the planned compositional model and generate interpretable estimates of reallocation effects, with precision assessed through confidence intervals. The present study's aim was achieved from 30‐min time reallocation effects estimated within an isometric log‐ratio (ILR)–transformed compositional data framework using quantile regression. These effects represent complex functions of multiple model coefficients, including interaction and higher‐order terms, rather than a single parameter. As such, their variance depends on the full variance–covariance structure of the compositional data, which cannot be specified reliably in advance. In addition, the use of median quantile regression introduces further complexity, as the asymptotic variance of parameter estimates depends on the unknown error density at the median.

### 2.7 Data Analysis

Data were analysed using R (R 4.2.1; R Core Team, 2023). Shapiro–Wilk tests were used to assess data distribution. Variables were expressed as count (percentages) for categorical data, mean ± SD if normally distributed, or median ± (25th–75th percentiles) for abnormally distributed continuous data. Sex differences between variables were explored using independent *t*‐test whilst pairwise Pearson correlation was used to assess the relationship between movement behaviours and cardiometabolic outcomes.

To evaluate the strength of emerging correlations, the effect of reallocating time between physical behaviours (sleep, SB, LPA, and MVPA) with another, on cardiometabolic parameters was estimated using compositional isotemporal substitution models [21]. First, each participant's raw minutes in sleep, SB, LPA, and MVPA were rescaled so that the four parts summed to 1 (i.e., proportions of a 24‐h day). Next, an isometric log‐ratio (ILR) transform was applied to the four‐part composition using default orthonormal basis in the R compositions package (sequential binary partition in column order: Sleep vs. [SB, LPA, and MVPA]; then SB vs. [LPA and MVPA]; then LPA vs. MVPA) [22]. To estimate a 30‐min substitution from behaviour A to behaviour B, we subtracted 30 min (30/1440 total daily proportion in minutes) from A and added 30 min to B (B = 1000), while holding other behaviours at their sample‐mean proportions. For each continuous outcome (e.g., VO_2_peak, HDL, and BMI), we fitted a median (*τ* = 0.5) quantile‐regression model, that is, Median̂ (***Y*** ∣ ILR(***x***), ***Z***) = ILR(***x***)^⊤**β**^+ ***Z*** ^⊤ ***Y***^, including all quadratic and pairwise interaction terms among the three ILR coordinates. Covariates (*Z*) were age, gender, employment status, height, and weight. Quantile regression was implemented with the Barrodale–Roberts algorithm (the rq(…, method = ‘br’) function in the R quantreg package), which is intrinsically robust to heavy‐tailed residuals [23]. We also trimmed any extreme ILR values to ensure strictly positive compositions prior to fitting.

To obtain confidence intervals for the predicted median outcome under each 30‐min reallocation, we used 95% percentile–bootstrap confidence intervals from adjusted median quantile regression models fitted on the original biomarker scale [24, 25].

## 3 Results

Anthropometric and clinical characteristics of participants are provided in Table 1. Briefly, 51% of the sample were female, with mean sample age of 40 ± 10 years (range 19–62). Most participants (88%) provided ≥ 3 days of monitor data and four participants who provided less than 24 h of data were excluded from movement behaviour analysis. Twenty‐six percent had elevated total cholesterol (> 5 mmol/L) and 84% with BMI ≥ 25 kg/m^2^.

**TABLE 1:** Descriptive characteristics of participants (*n* = 75).

Variable	All	Female	Male
Sociodemographic %			
Gender		52	48
Education			
Tertiary	85	48	52
Secondary	3	100	0
Unclassified	12	56	44
Risk status			
Non‐smoker	99	49	51
Diabetes	1	0	100
Employment			
Full‐time employed	56	53	48
Student	34	43	57
Unemployed	4	67	33
Unclassified	6	75	25
Anthropometrics	Mean ± SD		
Age (in years)	40 ± 10	40 ± 10	39 ± 9
Height (cm)**	171 ± 10	164 ± 7	178 ± 8
Waist circumference (cm)	93 ± 12	91 ± 12	94 ± 12
Weight (kg)	85 ± 16	82 ± 16	88 ± 14
Body mass index (kg/m^2^)	29.4 ± 5.6	30.7 ± 5.8	28.2 ± 5.3
Cardiometabolic	Mean ± SD		
Systolic blood pressure	125 ± 16	122 ± 17	128 ± 14
Diastolic blood pressure	79 ± 10	81 ± 11	77 ± 8
Total cholesterol (mmol/L)	4.3 ± 1.2	4.3 ± 1.2	4.3 ± 1.2
High‐density lipoprotein (mmol/L)*	1.5 ± 0.4	1.6 ± 0.4	1.3 ± 0.3
Fasting glucose (mmol/L)	5.9 ± 3.2	6.0 ± 4.1	5.8 ± 2.0
Low‐density lipoprotein (mmol/L)*	3.0 ± 1.1	2.7 ± 0.7	3.6 ± 1.1
Total cholesterol/high‐density lipoprotein*	3.1 ± 1.0	2.7 ± 1.1	3.3 ± 1.0
Peak oxygen consumption (mL/kg/min)**	18 ± 6	15 ± 3	20 ± 6
Movement behaviour categories!	Median ± IQR		
Wear time (minutes/day)	932 ± 139	931 ± 133	925 ± 153
Inactivity (minutes/day)	672 ± 139	679 ± 116	666 ± 159
LPA (minutes/day)	112 ± 46	114 ± 44	106 ± 56
MVPA (minutes/day)	96 ± 57	100 ± 44.9	107 ± 90
Sleep duration (minutes/day)	346 ± 102	347 ± 102	346 ± 110
Sleep efficiency (%)	76	77	76
Step count/day	10,074 ± 5120	9626 ± 3527	10,775 ± 6835

Abbreviations: IQR, interquartile range; LPA, light intensity physical activity; MVPA, moderate‐vigorous physical activity.

* *p* < 0.05

** *p* < 0.01; between male and female.

^!^ *n* = 71.

### 3.1 Association Between Movement Behaviours and Cardiometabolic Outcomes

**FIGURE 1:**
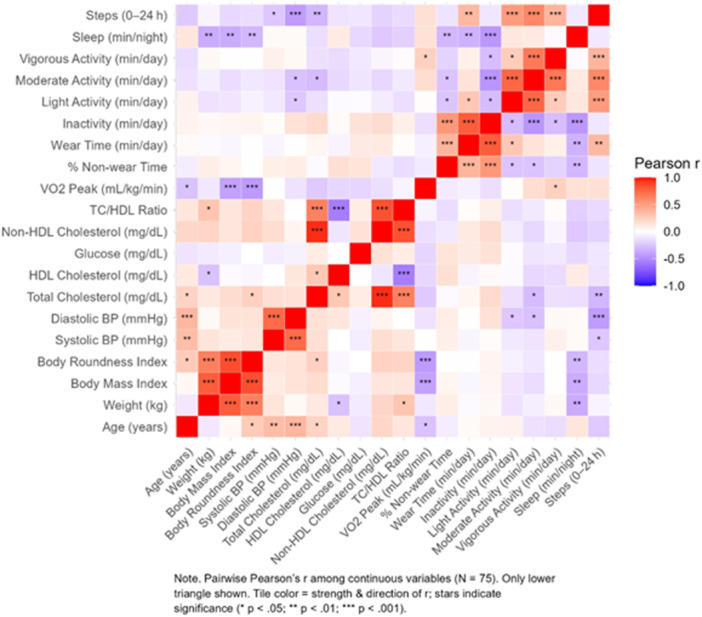
Heat map of pairwise correlations between movement behaviours and cardiometabolic markers.

Pairwise association was performed for all samples due to clinically unremarkable differences between males and females. Movement behaviours had no relationship with either blood glucose or blood lipids except for MVPA which showed a moderate inverse relationship with TC. There was no relationship between BMI or WC with any movement behaviour except sleep which showed a significant inverse relationship with BMI and WC (Figure 1). Step counts were also directly associated with lower TC. Only MVPA was associated with cardiorespiratory fitness mediated primarily via vigorous intensity activity.

### 3.2 Isotemporal Substitution of Movement Behaviours

**FIGURE 2:**
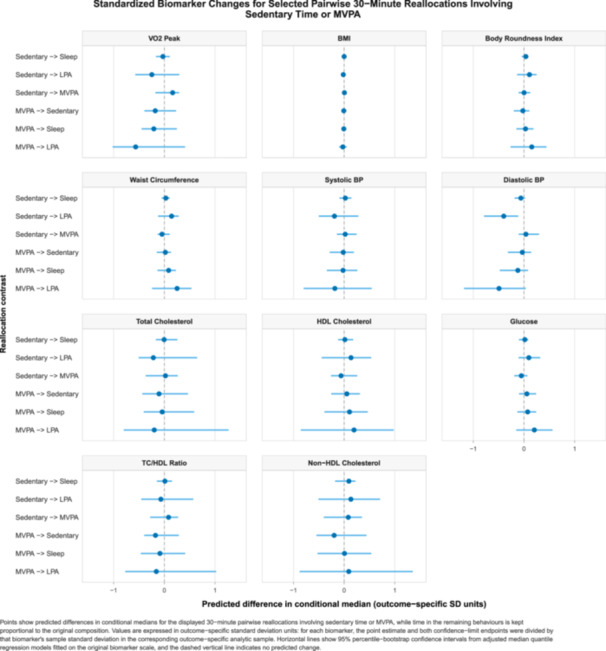
Standardised biomarker changes for selected pairwise 30‐min reallocations involving sedentary time or MVPA. Predicted difference in conditional median (outcome–specific SD units). Points show predicted differences in conditional medians for the displayed 30‐min pairwise reallocations involving sedentary time or MVPA, while time in the remaining behaviours is kept proportional to the original composition. Values are expressed in outcome–specific standard deviation units: for each biomarker, the point estimate and both confidence–limit endpoints were divided by that biomarker's sample standard deviation in the corresponding outcome–specific analytic sample. Horizontal lines show 95% percentile–bootstrap confidence intervals from adjusted median quantile regression models fitted on the original biomarker scale, and the dashed vertical line indicates no predicted change.

Replacing 30 min of SB with 30 min of either sleep, LPA, or MVPA showed no significant effect across all outcome measures (Figure 2). In addition, 30‐min reallocations of MVPA to other movement behaviours did not yield measurable cardiometabolic benefits (Figure 2).

## 4 Discussion

The present study examined cross‐sectional associations between device‐measured 24‐h movement behaviours and cardiometabolic health outcomes in individuals of Sub‐Saharan African heritage resident in the UK. To the best of our knowledge, this is the first study to assess movement behaviours in relation to a broad spectrum of cardiometabolic markers, including blood glucose, lipids and cardiorespiratory fitness. Analysis revealed that, among all 24‐h movement behaviours, only MVPA seemed to be associated with cardiometabolic markers: higher MVPA correlated inversely with total cholesterol and positively with peak oxygen consumption, a marker of cardiorespiratory fitness. Sleep duration exhibited an inverse correlation with BMI and body weight, suggesting a potential role in adiposity regulation. Neither sedentary behaviour nor light‐intensity physical activity demonstrated significant associations with any cardiometabolic outcome. Whilst these findings are informative, it is important to recognise that movement behaviours, that is, sleep, sedentary time, light physical activity, and moderate‑to‑vigorous physical activity are inherently compositional, as they collectively sum to a fixed 24‑h period. Consequently, correlations calculated on raw time‑use data may be influenced by the closure constraint, potentially leading to spurious associations. Compositional data analysis (CoDA) methods, including log‑ratio transformations, have been developed to appropriately account for the simplex geometry of time‑use data and to mitigate this issue. However, in the present study, correlations based on raw minutes were used deliberately as an exploratory and descriptive approach to facilitate clinical and public‑health interpretability. Log‑ratio–transformed values, while statistically appropriate, are not readily interpretable in real‑world units (e.g., minutes of activity or sleep), which may limit their usefulness for translational interpretation. Accordingly, Pearson correlations were employed to provide an intuitive overview of bivariate relationships rather than to support causal inference. These findings should therefore be interpreted cautiously and within the context of the compositional nature of 24‑h movement behaviours. More elaborate compositional reallocation analysis, which relied on increase in one movement behaviour whilst holding other behaviours constant revealed there were no significant cardiometabolic benefits from reallocating 30 min of sedentary time or MVPA to other movement behaviours.

Previous studies primarily conducted in White populations have consistently demonstrated strong positive association between PA and cardiometabolic health, unlike SB, currently regarded as a 21st century health concern [26] with significant links to several health conditions [11]. Increased MVPA has been shown to reduce total cholesterol, blood pressure, improve sleep quality, glucose levels, lower all‐cause mortality [12, 27, 28, 29, 30, 31], and improve quality of life [32]. Even a modest increase of 4–12 min of MVPA per day can yield clinically significant cardiometabolic improvements [8], likely mediated through complex interactions among inflammatory, haemodynamic, and metabolic pathways [7, 33, 34]. Whilst our findings seem to suggest limited cardioprotective benefits of increased MVPA primary via its influence of total cholesterol in individuals of Black heritage, these benefits may only be achieved via prolonged time exposures to this form of exercise. Additionally, the absence of a significant association between light PA and cardiometabolic outcomes contrasts with studies highlighting the potential benefits of light PA [35] or non‐exercise activity thermogenesis (NEAT) [36] on cardiometabolic health.

Previous literature highlighted strong associations between sleep duration and obesity [37]; our study uniquely identified an inverse relationship between sleep duration and BMI without observable links to other metabolic parameters such as lipid profiles or blood glucose. This could suggest a threshold effect, where only chronic sleep deprivation (rather than variations within normative ranges) substantially impacts metabolic homeostasis. However, these warrant further exploration, particularly in the context of lifestyle and environmental factors unique to individuals of SSA heritage.

Recent reports show interindividual variability in exercise response and whilst adherence to exercise prescription may yield general health benefits, targeted cardiometabolic risk factors may not improve [38]. This concept is especially important in Black populations, as inherent variations in cardiorespiratory fitness and fat oxidation capacity may play a role in shaping differences in cardiometabolic risk profiles [39], thus highlighting the need for unique PA recommendations in this population. Beyond MVPA, our findings align with previous research, which suggested limited cardiometabolic benefits from replacing SB with light PA [6, 40, 41], emphasising the importance of higher intensity PA [42] and potentially long‐term adherence coupled with other lifestyle modifications [43].

## 5 Limitations

The sample size, attributable to difficulty in recruiting participants from this population and cross‐sectional design of this study limits the ability to make causal inferences. Another limitation of this study is that a subset of participants attended the research facility around midday, which precluded objective verification of fasting status at the time of assessment. Although participants were instructed to fast prior to testing, variation in appointment timing may have introduced residual measurement variability in fasting‐dependent biomarkers.

Furthermore, isotemporal substitution modelling does not accurately capture real‐world behavioural time redistribution. Nevertheless, the inclusion of bootstrap‐percentile intervals alongside point estimates enhances the robustness of our inferences, even within the constraints of a modest sample size [44]. The null results with point estimates and 95% delta‐method confidence intervals define plausible bounds for 30‐min reallocations in a real‐world sample. Further prospective interventions that can explore longitudinal effects of behaviour are warranted to confirm our results.

## 6 Conclusion

This study is the first to show the relationship between objectively assessed movement compositions and markers of cardiometabolic health (including cardiorespiratory fitness) in a healthy but at risk of cardiovascular disease cohort of Black adults from SSA. MVPA may contribute to better cardiometabolic health via reduction in total cholesterol and improvement in cardiorespiratory fitness, but the frequency and volume required to elicit these changes in this population, may exceed current global physical activity guidelines. Our findings have substantial research and clinical implications, as it underscores the importance of promoting higher intensity and volume of MVPA in public health initiatives for Black populations while recognising the limited impact of SB reallocation to lower‐intensity movement. Further research is warranted to explore the role of other factors such as diet, socioeconomic conditions, and access to healthcare in determining overall health outcomes in this demographic.

## Author Contributions

**Damilola A. Ibirogba:** investigation, methodology, formal analysis, data curation, writing – original draft, writing – review and editing, project administration. **Luis S. Andalco:** methodology, data curation, formal analysis, writing – original draft, writing – review and editing, investigation, software. **Matteo Crotti:** conceptualisation, methodology, formal analysis, writing – original draft, writing – review and editing, project administration, software. **Sarah J. Charman:** conceptualisation, data curation, formal analysis, supervision, writing – original draft, writing – review and editing, project administration. **Amy S. Fuller:** methodology, data curation, formal analysis, writing – review and editing, investigation. **Titilope Ajepe:** methodology, formal analysis, investigation, writing – original draft, writing – review and editing. **Michael Duncan:** conceptualisation, data curation, investigation, supervision, writing – original draft, writing – review and editing, project administration. **Alasdair P. Blain:** methodology, data curation, formal analysis, investigation, writing – review and editing, software. **Faatihah Niyi‐Odumosu:** writing – review and editing, investigation, methodology, formal analysis. **Peter W. Mwangi:** conceptualisation, supervision, writing – original draft, writing – review and editing, methodology, data curation. **Federick O. Bukachi:** methodology, supervision, writing – review and editing, writing – original draft. **Olufumilola L. Dominic:** methodology, investigation, formal analysis, writing – review and editing. **Otto F. Barak:** methodology, writing – review and editing, data curation, investigation. **Djordje G. Jakovljevic:** conceptualisation, writing – review and editing, supervision, investigation, methodology, project administration. **Nduka C. Okwose:** conceptualisation, investigation, methodology, data curation, supervision, formal analysis, visualisation, funding acquisition, writing – original draft, writing – review and editing, project administration.

## Disclosure

All authors have read and approved the final version of the manuscript. N. C. Okwose had full access to all of the data in this study and takes complete responsibility for the integrity of the data and the accuracy of the data analysis. N. C. Okwose affirms that this manuscript is an honest, accurate, and transparent account of the study being reported; that no important aspects of the study have been omitted; and that any discrepancies from the study as planned (and, if relevant, registered) have been explained.

## Conflicts of Interest

The authors declare no conflicts of interest.

## Data Availability

The data that support the findings of this study are available from the corresponding author upon reasonable request.
